# Pharmacological and Pathological Implications of Sigma-1 Receptor in Neurodegenerative Diseases

**DOI:** 10.3390/biomedicines13061409

**Published:** 2025-06-08

**Authors:** Noah Drewes, Xiangwei Fang, Nikhil Gupta, Daotai Nie

**Affiliations:** Department of Medical Microbiology, Immunology and Cell Biology, Southern Illinois University School of Medicine, P.O. Box 19626, Springfield, IL 62794-9626, USA; ndrewes45@siumed.edu (N.D.);

**Keywords:** sigma-1 receptor, sigma receptor, TMEM97, neurodegenerative disorders, ER stress, Alzheimer’s disease, amyotrophic lateral sclerosis

## Abstract

Originally identified as a potential receptor for opioids, the sigma-1 receptor is now recognized as an intracellular chaperone protein associated with mitochondria-associated membranes at the endoplasmic reticulum (ER). Over the past two decades, extensive research has revealed that the sigma-1 receptor regulates many cellular processes, such as calcium homeostasis, oxidative stress responses, protein folding, and mitochondrial function. The various functions of the sigma-1 receptor highlight its role as a central modulator of neuronal health and may be a promising pharmacological target across multiple neurodegenerative conditions. Herein, we provide an overview of the current pharmacological understanding of the sigma-1 receptor with an emphasis on the signaling mechanisms involved. We examine its pathological implications in common neurodegenerative diseases such as Alzheimer’s disease, Parkinson’s disease, amyotrophic lateral sclerosis, Huntington’s disease, and multiple sclerosis. We then highlight how sigma-1 receptor modulation may influence disease progression as well as potential pharmacological mechanisms to alter disease outcomes. The translational potential of sigma-1 receptor therapies is discussed, as well as the most up-to-date results of ongoing clinical trials. This review aims to clarify the therapeutic potential of the sigma-1 receptor in neurodegeneration and guide future research in these diseases.

## 1. Introduction

Sigma receptors were first identified in the 1970s during investigations into the mechanisms of opioid action. W.R. Martin observed that SKF-10047, a derivative of morphine, caused mydriasis, tachypnea, tachycardia, and mania, distinct from those induced by known opioid receptor agonists such as morphine, a mu receptor agonist, and ketocyclazocine, a kappa receptor agonist [1]. These observations suggested that SKF-10047 was binding to an unrecognized receptor, as opposed to the traditional mu, kappa, and delta receptor subtypes. The “sigma” classification, derived from the Greek letter σ, is used to denote its distinct binding properties [1]. This discovery initiated considerable interest in identifying novel pathways through which the opioid-like compounds could exert their effects.

The 1980s and 1990s marked a turning point for sigma receptor research. Studies by Tam and Cook in 1984 demonstrated that the effects of sigma ligands could not be reversed by naloxone, an opioid antagonist, which is a defining feature of opioid receptors [2]. This observation led to the recognition that sigma receptors are pharmacologically distinct from opioid receptors due to their unique binding sites, affinities, pharmacological profile, and naloxone insensitivity [3].

Later, based on pharmacological profiles, binding characteristics, and tissue distribution patterns, sigma receptors were categorized into the sigma-1 receptor (S1R) and the sigma-2 receptor (S2R) [4]. For instance, S1R exhibits a high affinity for (+)-pentazocine, whereas S2R does not. In contrast, other ligands show preferential binding to S2R. These distinctions, paired with differences in molecular weight and other biochemical properties, laid the groundwork for distinguishing between the two receptor subtypes.

The cloning of S1R in 1996 by Hanner et al. marked a significant milestone, providing insight into its molecular structure and function [5]. The gene for S1R encodes a protein that differs from the traditional G protein-coupled receptors and instead shares some characteristics with molecular chaperones. In contrast, the molecular identity of the S2R remained elusive until 2017, when it was identified as a distinct entity from S1R and was shown to be involved in different cellular processes [6], representing another significant advancement in sigma receptor research.

Initially mistaken as a subtype of opioid receptors, sigma receptors have emerged as a distinct class of proteins with diverse roles in cellular signaling, neurophysiology, and pharmacology. Their discoveries have opened new avenues for understanding disease mechanisms and developing novel therapeutic strategies.

To address the growing interest in S1R-related therapeutics in neurodegeneration, we conducted a scoping review of the literature using a structured PubMed search. The review focused on articles published in the last decade (2015–2025). Terms that were searched included “sigma receptor,” “σ-1 receptor,” “σ-2 receptor,” among other related variations of the term. Emphasis was placed on mechanistic and translational studies.

**Figure 1:**
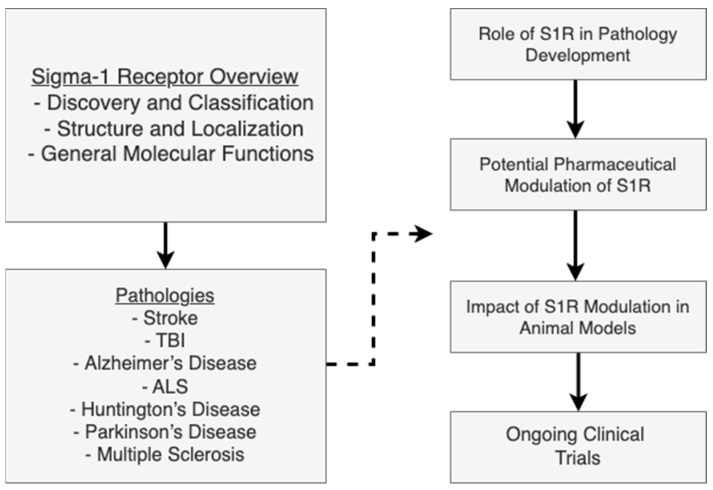
Structural framework of the review: from S1R biology to therapeutic application in neurological disease. Abbreviations: S1R, Sigma-1 receptor; TBI, traumatic brain injury; ALS, amyotrophic lateral sclerosis.

Given the broad functions of S1R and its numerous implications in pathological states, the structure of the review is split into diseases. We begin by exploring the structure and biology and move forward to pathologies. Of the pathologies, mechanical insults to the nervous system are first discussed. We subsequently review the implications of several neurodegenerative diseases. Each section reviews the implications of S1R in the development of these various pathological states, how pharmaceuticals may modulate this process using S1R, and the most up-to-date research, ranging from animal models to clinical trials. A schematic of the review is represented in Figure 1. Additionally, we provide an updated review of the literature with the newest results in the past five years.

## 2. Sigma-1 Receptor: Structure, Functions, and Pharmacology

S1R is a novel protein localized to the endoplasmic reticulum (ER), where it associates with lipid rafts [1,7,8,9]. The first cloning took place in 1996 and localized the gene to chromosome 9p13 [5,10]. S1R was later resolved structurally as a homo-trimer with a unique transmembrane domain for each region [5,11,12,13,14,15]. Its oligomerization state is modulated by ligand binding, as agonists promote multimer formation, whereas antagonists reduce it [16]. S1R is additionally capable of forming heteromeric complexes with other receptors, such as the dopamine receptor [17,18]. With a molecular weight of 25 kDa, S1R is structurally unique and shares no homology to other known proteins in the genome [9,19].

Following the identification of S1R, S2R was described, though it remains less characterized. Like S1R, S2R binds to SKF-10047 and associates with lipid rafts [7,20,21,22]. Additional research has shown that S2R is potentially a binding site for progesterone receptor membrane component 1 (Pgrmc1) [23] and was later identified as the known transmembrane protein TMEM97 [6]. Currently, S1R and S2R are primarily distinguished based on ligand binding assays. H3(+) pentazocine has a selective affinity for S1R, whereas H3-DTG or H3-(+)-3-PPP combined with a masking S1R ligand, such as pentazocine, is used to selectively label S2R [24,25].

S1R plays many roles in cellular biology. It is capable of translocating between the ER, mitochondria, and plasma membrane [26,27,28,29]. In addition, S1R interacts with G-proteins, ion channels, inositol 1,4,5-triphosphate (IP3) receptors, and glutamate receptors, mediating diverse signaling pathways [29,30,31]. It is widely expressed in multiple vital organs, such as the heart, liver, and kidney, as well as in immune cells [32]. Pharmacologically, S1R binds a wide variety of ligands, such as antipsychotics, antidepressants, and neurosteroids [3,33]. Currently, it has been implicated in cell survival, as its expression is elevated in various cancers. Furthermore, S1R acts as a molecular chaperone with established roles in neuroprotection [8,34,35,36,37].

**Figure 2:**
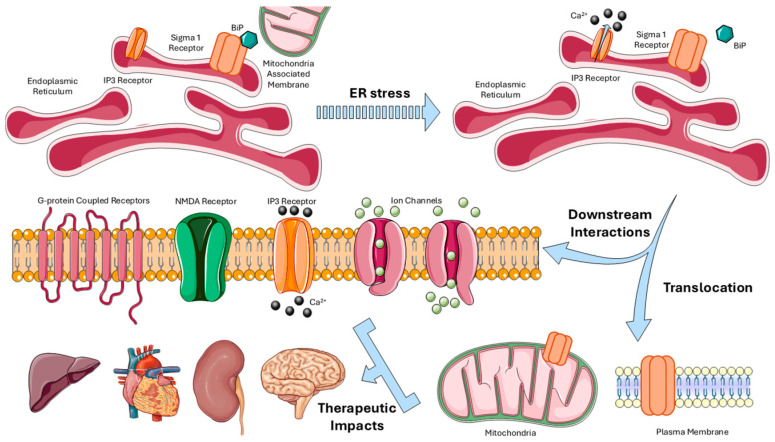
S1R localization, activation, and downstream interactions. Under basal conditions, S1R resides at the mitochondria-associated endoplasmic reticulum membrane (MAM), tethered to BiP. Upon ER stress, S1R dissociates from BiP and binds to inositol 1,4,5-triphosphate receptors, facilitating calcium signaling between the ER and mitochondria. Activated S1R translocates to other compartments, such as the plasma membrane. S1R can modulate ion channels, NMDA receptors, and G-protein coupled receptors. These downstream interactions mediate calcium homeostasis as well as reductions in ER dysfunction and oxidative stress. S1R is implicated in therapeutic benefits for the brain, heart, liver, and kidney. Abbreviations: S1R, Sigma-1 receptor; MAM, mitochondria-associated membrane; BiP, binding immunoglobulin protein; ER, endoplasmic reticulum; IP3, inositol 1,4,5-triphosphate; NMDA, N-methyl-D-aspartate. Figure created using Servier Medical Art (CC BY 3.0 license).

In the resting state, S1R is localized to mitochondria-associated ER membranes (MAM), where it resides in ceramide- and cholesterol-rich microdomains with the ER chaperone BiP [27,38]. Under times of cellular stress, ER dysfunction prompts S1R to dissociate from BiP. Upon dissociation, S1R binds IP3 receptors, enhancing cell survival through calcium signaling between the ER and mitochondria. Previous testing has shown that S1R agonists amplify this stress response while antagonists inhibit it [27]. An overview of the structure and function of S1R is represented in Figure 2.

Overall, under physiological conditions, S1R plays a minor role but transitions to an active chaperone protein in times of stress, supporting cell survival [30]. Aside from the IP3 pathway, S1R modulates dopaminergic and cholinergic transmission by influencing associated ion channels [39,40,41,42].

## 3. Sigma-1 Receptor in Nerve Injury

Nervous system injuries can result from a range of insults, including mechanical trauma and ischemia. These can affect both central and peripheral structures [43,44,45]. Following nerve injury, S1R expression has been shown to increase [46]. In ischemic stroke, S1R is hypothesized to exert neuroprotective effects by preventing neural apoptosis and inflammation while promoting neurotrophic signaling [47,48]. A primary driver of apoptosis following ischemic stroke includes endoplasmic reticulum stress, which can be attenuated by S1R activation with dexmedetomidine or aniline derivatives by reducing ER stress proteins CHOP, Caspase-3, and phosphorylated-JNK [48,49].

Strokes remain one of the leading causes of disability worldwide, with ischemic events composing the majority [50]. Generally, the prognosis of a stroke often correlates with the extent of ischemia, representing the amount of irreversibly damaged neural tissue [51]. Preclinical studies with the S1R agonists N,N-Dimethyltryptamine (DMT), and oxeladin have demonstrated potential in reducing the size of an infarct and improving long-term outcomes. DMT additionally reduced APAF1 levels, a marker of apoptosis, as well as inflammatory markers TNF-α, IL1-β, and IL-6, while increasing BDNF levels, central to neural preservation. Early-phase clinical trials for ischemic stroke in healthy individuals confirmed the safety of varying doses of DMT, opening avenues for future treatments [47,52,53,54]. Other markers of apoptosis, such as Bax and caspase-3, were markedly reduced in microglial cells following a stroke when treated with the S1R agonist afobazole [55]. Reactive gliosis following ischemic stroke is a maladaptive reaction that scars the brain parenchyma [56]. The S1R agonist afobazole, as well as the S2R antagonist S1RA, reduced the number of reactive cells through a reduction in matrix metalloproteinase-9, contributing to better recovery [55,57]. Aside from limiting acute injury, S1R activation through the selective agonist PRE-084 promoted recovery of white matter injury, decreased demyelination, and increased myelination factors CNPase, MOG, and PDGFRα after a stroke in preclinical models [58]. Following an ischemic stroke, macrophage-mediated efferocytosis is critical for clearing cellular debris from the infarct and inducing neural repair and inflammation resolution. Recent studies show that S1R knockout (KO) models impair this process and exacerbate brain damage following an ischemic stroke [59,60]. The benefits of S1R agonism following stroke are not limited to motor recovery but also suggest cognitive improvement in post-stroke models when treated with the S1R agonists ulinastatin, oxeladin, and PRE-084 by rescuing BDNF through the NR2A-CaMKIV-TORC1 pathway [52,61,62,63]. Neurite outgrowth is another central mechanism to post-stroke recovery, which was induced with the S1R agonist TS-157 through the upregulation of phosphorylated ERK [64].

Stroke-induced damage also compromises blood–brain barrier (BBB) integrity and increases permeability to substances otherwise restricted from the brain [65]. S1R agonism with PRE-084 and dexmedetomidine successfully attenuated this damage by inducing BBB stability through increasing levels of glia-derived neurotrophic factor (GDNF), restoring abnormal expression of the BBB marker Occludin, and decreasing inflammatory markers [58,62,66,67]. Pericyte attachment acts as another contributor to BBB breakdown, which has been shown to improve with S1R activation [68,69]. Post-stroke complications include spreading depolarizations (SDs), which are waves of depolarizations from neural cells due to a failure of ion homeostasis after an insult [70,71]. PRE-084 and DMT have been shown to reduce SD frequency, promoting neural survival and reducing apoptosis [72]. Another feared complication of ischemic stroke includes reperfusion injury, characterized by a paradoxical worsening of damaged cells upon restoration of blood flow, causing inflammation and apoptosis. S1R upregulation appears to attenuate this process and improve outcomes [63,73].

In models of traumatic brain injury (TBI), the role of S1R is complex. In one study, mice deficient in sigma receptors led to better long-term neurological outcomes, such as better coordination and fewer neurological deficits after one-year post-injury [74]. Conversely, other investigations of TBI demonstrate that S1R agonism leads to better neurological function, restoration of blood flow, and decreased brain edema [75]. This apparent biphasic effect may reflect differing impacts of S1R activation depending on timing, with acute-phase activation being beneficial and prolonged activation being potentially detrimental. The acute phase of TBI is marked by inflammation, which is dampened with S1R activation [76]. Although the role of S2R in TBI is less studied, modulation may yield neuroprotective outcomes [77].

**Figure 3:**
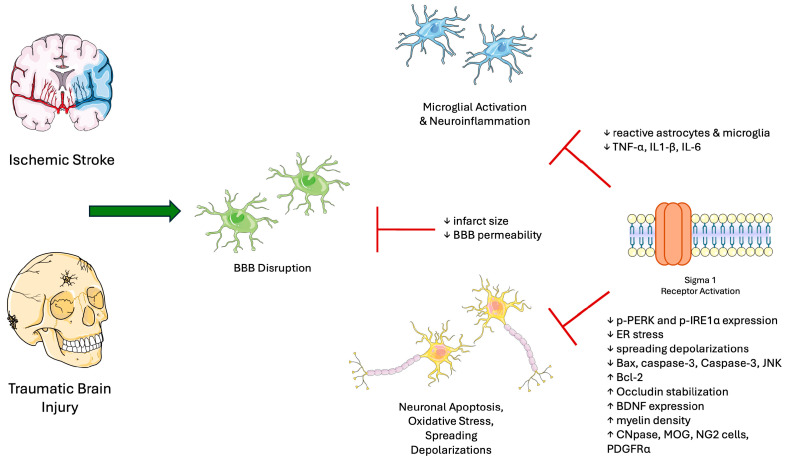
Role of S1R in nervous system injury. Events such as ischemic stroke and TBI trigger a cascade of processes such as BBB breakdown, microglial activation, and neuronal apoptosis. These are mediated through ER stress, spreading depolarizations, and neuroinflammation. Activation of S1R reduces these mechanisms and promotes neuroprotection. Several S1R modulators have been studied in preclinical models and demonstrate beneficial effects. Abbreviations: S1R, Sigma-1 receptor; TBI, traumatic brain injury; BBB, blood–brain barrier; ER, endoplasmic reticulum; IL1-β, interleukin-1 beta; IL-6, interleukin-6; TNF-α, tumor necrosis factor alpha; p-PERK, phosphorylated protein kinase RNA-like ER kinase; p-IRE1α, phosphorylated inositol-requiring enzyme 1 alpha; JNK, c-Jun N-terminal kinase; BDNF, brain-derived neurotrophic factor; CNPase, 2′,3′-cyclic-nucleotide 3′-phosphodiesterase; MOG, myelin oligodendrocyte glycoprotein; PDGFRα, platelet-derived growth factor receptor alpha. Figure created using Servier Medical Art (CC BY 3.0 license).

In the context of spinal cord injury, S1R activation has demonstrated protective effects in recovery after a mechanical insult to the spine. This was achieved through reducing neuroinflammation, apoptosis, and ferroptosis [78,79]. Ferroptosis is an iron-dependent form of cell death characterized by the buildup of lipid oxidation products. Ferroptosis has been implicated in neuronal cell death after injury and is shown to be upregulated in the setting of spinal cord injury; however, treatment with DMT resulted in marked reductions in this process [72,78,80,81]. The impact of neural injury and the role of S1R is represented in Figure 3.

## 4. Sigma-1 Receptor in Neurodegenerative Disorders

Sigma receptors have been implicated in a variety of neurodegenerative disorders due to their role in regulating calcium homeostasis, mitochondrial function, and oxidative stress regulation [29,82,83,84]. Genetic polymorphisms in the S1R gene have been associated with increased susceptibility to Alzheimer’s disease (AD), whereas loss-of-function mutations have been linked to the development of amyotrophic lateral sclerosis (ALS) and frontotemporal dementia (FTD), potentially through mechanisms such as impaired long-term potentiation observed in S1R KO models [85,86,87,88]. Other neurological disorders have had favorable outcomes with sigma-receptor modulation, such as Huntington’s disease (HD), multiple sclerosis (MS), and Parkinson’s disease (PD) [89,90,91]. In recent years, S1R has gained attention as a promising therapeutic target for mitigating these diseases [92]. Additionally, S2R ligands are also emerging as potential modulators of neurodegeneration [93].

S1R agonism confers neuroprotection through several pathways. These include enhanced synaptic plasticity through brain-derived neurotrophic factor (BDNF) dependent mechanisms, reduction of intracellular nitric oxide (NO) by inhibiting NO synthase, and prevention of oxidative stress by reducing the accumulation of reactive oxygen species (ROS) [94,95,96,97,98,99,100,101]. All of these mechanisms support the maintenance of homeostatic plasticity, a process critical to the stabilization of neural pathways, and are central to preventing neurodegeneration [102,103]. Functional evidence of neuroprotection with S1R agonism has been studied in behavioral mouse models using modalities such as novel object recognition, showing improved cognitive outcomes [104,105].

Neuroinflammation also plays a central role in the progression of neurodegenerative disorders [106]. Downregulation of S1R has been associated with increased inflammation markers and disrupted microglial homeostasis [107]. In contrast, S1R agonists and allosteric modulators have been shown to reduce neuroinflammation levels through the reduction of microglial recruitment and inhibition of pro-inflammatory cytokine production, thereby attenuating gliosis and slowing cognitive impairment from various degenerative conditions, such as chronic epilepsy [108,109,110,111].

### 4.1. Sigma-1 Receptor in Alzheimer’s Disease

Mitochondrial dysfunction is a hallmark of many neurodegenerative disorders, including Alzheimer’s disease (AD) [112]. Restoring mitochondrial stability has become a major therapeutic goal with S1R modulation [113]. In preclinical models, DMT preserved mitochondrial integrity by restoring the expression of MAM-associated proteins. Chronic treatment with DMT not only led to general neuroprotection but also slowed the accumulation of beta-amyloid in the hippocampus, the pathological hallmark of AD [114,115,116,117,118,119]. Similar benefits have been observed with pridopidine and PRE-084, which restored mitochondrial dysfunction by reducing levels of ROS and subsequent astrogliosis as well as stimulating hippocampal proliferation [89,116,118,119,120,121,122,123]. Furthermore, S1R agonism demonstrated synergistic neuroprotective effects when combined with the acetylcholinesterase inhibitor donepezil, the current treatment for AD, again through ROS reduction [100,124].

Cognitive impairment in AD is commonly tested through recognition memory in animal models, which have shown improvement after treatment with the S1R agonists DMT, AF710B, pridopidine, OZP002, LS-1-137, and WLB-87848. AF710B reduced the number of amyloid plaques as well as astrogliosis in the hippocampus. Pridopidine induced the formation of neuronal synapses through activation of the ERK and Akt pathways. OZP002 reduced ROS, apoptotic marker Bax, inflammatory markers TNFα and IL-6, and lipid peroxidation within hippocampal neurons [108,114,117,122,125]. Other S1R agonists, such as dipentylammonium (DPA), did not significantly alter cognitive outcomes but were found to induce neurite growth through increasing levels of NGF and improved lifespan in preclinical models [126,127]. However, some studies have reported paradoxical findings. Specifically, MAM induction has been linked to increased amyloid-beta accumulation, which was reduced with S1R downregulation [128].

Another hallmark of AD pathogenesis is the deposition of neurofibrillary tangles composed of hyperphosphorylated tau protein [129]. In healthy individuals, S1R has been implicated in maintaining proper phosphorylation of tau proteins, and loss of S1R may contribute to tau-related pathology [130,131]. S2R modulation also has a potential role in slowing the progression of AD. CT1812 and SAS-0132, S2R antagonists, displaced amyloid plaques from hippocampal neurons, induced synapses, and regulated tau phosphorylation, improving cognitive performance. WLB-8962, an S2R agonist, improved short-term memory in preclinical models [132,133,134]

**Figure 4:**
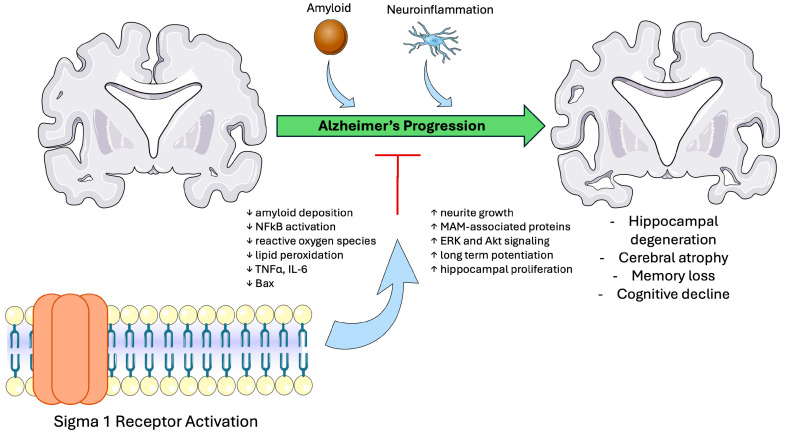
Proposed protective mechanisms and pharmaceutical implications of sigma-1 receptor in Alzheimer’s disease. This schematic illustrates the progression of AD driven by the deposition of amyloid plaques in the nervous system. S1R activation modulates several key processes in AD pathogenesis to slow cognitive decline and disease progression. Abbreviations: S1R, Sigma-1 receptor; AD, Alzheimer’s disease; NFkB, nuclear factor kappa B; TNFα, tumor necrosis factor alpha; IL-6, interleukin-6; Bax, Bcl-2-associated X protein; MAM, mitochondria-associated membrane; ERK, extracellular signal-regulated kinase; Akt, protein kinase B. Figure created using Servier Medical Art (CC BY 3.0 license).

Other mechanisms under investigation to slow the progression of AD include the role of preserving BBB integrity. S1R activation has been associated with increasing levels of vascular endothelial growth factor (VEGF) and low-density lipoprotein receptor-related protein 1 (LRP-1), both of which are critical to maintaining BBB function [135]. Newly studied S2R ligands have also demonstrated promising results in beta-amyloid-induced neurological dysfunction, potentially through restoration of calcium homeostasis [133,134]. Figure 4 demonstrates the role of S1R in slowing the progression of AD.

Ongoing clinical trials utilizing S1R or S2R ligands offer promising results for the future of AD treatment. A recent Phase IIb/III trial was conducted in patients with AD to test the efficacy of blarcamesine, an S1R agonist. Blarcamesine significantly slowed cognitive deterioration by 36.3% compared to placebo after 48 weeks. Those with the SIGMAR-1 genotype exhibited the most improvement [136]. Another S1R agonist, T-817MA, was studied in a Phase II trial but did not demonstrate statistically significant reductions in cognitive decline [137]. CT1812 is currently undergoing multiple Phase I and II trials, and a systematic review revealed that Phase I trials demonstrate adequate drug safety and cerebral penetration, opening avenues for future trials to assess their disease-modifying capabilities [138].

### 4.2. Sigma-1 Receptor in ALS

ALS is a devastating neurodegenerative disease, with the most common inherited form linked to mutations in the C9orf72 gene involving repeats of GGGGCC-RNA [139]. MAM instability and dysregulated autophagy are key pathological features of ALS [140,141,142]. Notably, a common selective serotonin reuptake inhibitor (SSRI), fluvoxamine, possesses S1R agonism and has been shown to restore autophagic balance in inherited ALS by stabilizing nucleoporin complexes with Pom121 protein expression and localizing the autophagy factor TFEB to the nucleus [143].

Additional S1R agonists such as pridopidine, PRE-084, and SA4503 have demonstrated improvements in motor behavior and neuroprotection of ALS mouse models [144,145]. PRE-084, specifically, rescued the mitochondrial dysregulation and ER stress seen in ALS, primarily through increasing the ATF4 and NRF2 antioxidant cascades [121]. Interestingly, in the same experimental settings, BD1063, an S1R antagonist, also yielded neuroprotective effects through a similar mechanism to PRE-084, suggesting a more complex role of S1R signaling in ALS [145].

Another pathological mechanism contributing to the development of ALS involves the dysregulated accumulation of repeat-associated non-AUG (RAN) proteins within neurons. Overexpression of S1R has been shown to reduce RAN protein accumulation, offering a novel therapeutic avenue [146,147]. Additionally, mutations in the Cu/Zn superoxide dismutase (SOD1) gene, another hallmark of familial ALS, can lead to neurofilament accumulation and motor neuron degeneration. Treatment with pridopidine reduced this buildup by activating the ERK pathway, highlighting its potential as a disease-modifying therapy [148,149]. Pridopidine is presently the only sigma receptor ligand undergoing clinical trials for ALS. In a Phase II/III trial studying pridopidine’s ability to alter functional outcomes, respiratory status, and survival in ALS, no significant differences were noted when compared to placebo after 24 weeks [150].

### 4.3. Sigma-1 Receptor in Huntington’s Disease

HD is an inherited neurodegenerative disorder characterized by progressive neuronal loss, leading to debilitating, uncontrolled movements [151]. As with many neurodegenerative diseases, mitochondrial dysfunction is central to HD pathogenesis [152]. In preclinical models of HD, pridopidine restored the antioxidant defenses and reduced levels of mitochondrial reactive oxygen species through rescuing IP3R localization as well as BDNF, glucocorticoid receptor (GR) dopamine D1 receptor (D1R), cAMP, and TrkB signaling, which restored glutamate homeostasis within the striatum [89,94,153,154]. Pridopidine also restored ER stress through the reduction in the unfolded protein response, which is central to the pathogenesis of HD, most prominently in the PKR-like endoplasmic reticulum kinase (PERK) pathway [155,156]. Additional studies have demonstrated that pridopidine restores calcium homeostasis through the upregulation of calbindin and homer1a, contributing to the attenuation of disease progression [154,156,157,158].

Given that there are currently no approved disease-modifying therapies for HD, the development of novel treatment modalities is urgently needed. There are multiple ongoing clinical trials investigating pridopidine in the treatment of HD. In a review investigating four trials, all in Phase II or III with 1067 total patients with HD, pridopidine was shown to be safe in all cohorts, offering promising treatment modalities for the future [159,160].

### 4.4. Sigma-1 Receptor in Parkinson’s Disease

PD is one of the most prevalent neurodegenerative disorders, characterized by bradykinesia, tremor, and rigidity [161]. A key pathological feature is the toxic accumulation of alpha-synuclein in the nervous system, which contributes to mitochondrial dysfunction and subsequent neurodegeneration [162]. Deficiencies in S1R exacerbate alpha-synuclein accumulation, highlighting its importance in the pathogenesis of PD [163].

In addition, S2R antagonists have demonstrated neuroprotective effects by mitigating alpha-synuclein-induced toxicity, suggesting a novel therapeutic modality [164]. S1R agonism through PRE-084, either alone or in combination with nicotinic receptor agonists, has shown efficacy in preserving dopaminergic neurons, which are preferentially impacted in PD, as well as providing neuroprotection from MPTP-induced neuronal damage [165,166,167]. Pridopidine was found to improve motor deficits in preclinical models of PD after long-term treatment by increasing the number of dopaminergic neurons in the striatum through upregulation of GDNF, BDNF, and phosphorylated ERK [167]. Levodopa is a mainstay treatment of PD; however, it is frequently associated with dyskinesia as a side effect [168]. Preclinical studies have shown that pridopidine may attenuate levodopa-induced dyskinesia while preserving the benefit [169,170].

**Figure 5:**
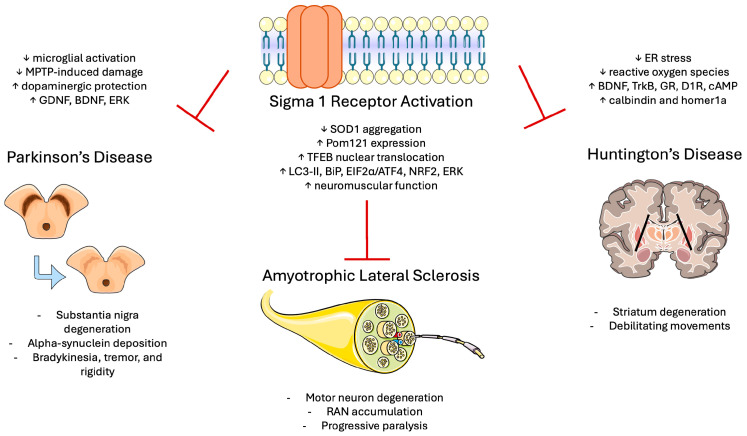
Disease-specific pathways of Sigma-1 receptor activation in various neurodegenerative diseases. In PD, S1R activation protects dopaminergic neurons and counteracts alpha-synuclein accumulation. In ALS, S1R reduces SOD1 and RAN accumulation, preventing the loss of motor neurons. In HD, S1R helps prevent degeneration of the striatum by relieving ER stress and stimulating neurotrophic proteins. Abbreviations: S1R, Sigma-1 receptor; GDNF, glial cell-derived neurotrophic factor; BDNF, brain-derived neurotrophic factor; ERK, extracellular signal-regulated kinase; GR, glucocorticoid receptor; D1R, dopamine D1 receptor; cAMP, cyclic adenosine monophosphate; TFEB, transcription factor EB; RAN, repeat-associated non-AUG. Figure created using Servier Medical Art (CC BY 3.0 license).

S1R antagonists have also demonstrated a protective effect on PD pathogenesis. One study found that S1R downregulates the transient receptor potential canonical (TRPC) channel, which is critical for calcium signaling and maintaining cell viability. S1R antagonism in this context restored TRPC activity and promoted dopaminergic neuroprotection [171]. Additionally, reduced S1R expression has been associated with decreased NMDA-receptor-mediated excitotoxicity, further supporting its complex role in PD [172]. The implications of sigma receptors in modifying the disease state of ALS, HD, and PD are demonstrated in Figure 5.

### 4.5. Sigma-1 Receptor in Demyelinating Disorders

Demyelinating disorders, both inherited and acquired, have also been investigated in the context of S1R agonism. Krabbe Disease, a rare autosomal recessive leukodystrophy marked by progressive neurodegeneration and demyelination, has shown positive responses to S1R modulation. In animal models, treatment with donepezil, which possesses S1R agonism, preserved myelin integrity and reduced glial reactivity [173,174]. Similar results have been reported in other rare genetic neurodegenerative disorders, such as Wolfram Syndrome and Vanishing White Matter Disease (VWM), where S1R activation demonstrated protection against cellular stress and mitochondrial dysfunction, lowering rates of autophagy [175,176,177,178].

In contrast to these inherited conditions, MS is an acquired autoimmune disease characterized by chronic inflammation, demyelination, and axonal loss [179]. Preclinical studies investigating models of MS concluded that S1R agonists can attenuate the clinical course, offering a promising avenue for clinical translation [110,180]. Specifically, blarcamesine protected oligodendroglia from ROS and quinolinic acid, a common neuroinflammatory marker [90]. The neuroprotective mechanism in MS appears to involve the preservation of oligodendroglia from apoptosis and reactive oxygen species [90]. Presently, there are no current sigma receptor ligands in the clinical stage for demyelinating disorders.

## 5. Challenges and Future Directions

Despite the significant advances in understanding S1R and its role in neurodegenerative diseases, several challenges limit its clinical translation. Though many mouse models offer promising results in attenuation of neurodegeneration, the precise pathways and downstream targets behind them remain less understood. This significantly limits the ability to evaluate these medications on patients. Additionally, many ligands that have been developed for S1R are rarely selective and may be modulating other pathways that remain undiscovered. The variability in results adds to the difficulty in assessing S1R as a promising target. This adds the need for more context-dependent studies. It will be critical to elucidate the S1R function at various disease stages as well as its tissue-specific expression. The lack of clinical trials, specifically beyond the early phases, leaves S1R undiscovered in the context of its ability to slow neurodegeneration in humans. Despite the progress made, it is imperative to further evaluate the utility of these ligands in a clinical setting.

## 6. Conclusions

In conclusion, sigma receptors have emerged as key regulators of cellular homeostasis and neuroprotection. Table 1 summarizes key S1R and S2R ligands currently under investigation, including their target diseases, mechanisms, and stage of clinical development. Their broad expression across tissues has driven extensive research into their pharmacologic potential, particularly within the nervous system. Their versatile roles across a variety of pathologies emphasize their relevance as novel therapeutic targets in future research for both acute and chronic neurodegeneration. However, contradictory findings in different models highlight the importance of temporal and tissue-specific modulation to optimize outcomes. Additionally, the integration of S1R into combination therapies may enhance clinical benefit, offering new strategies for previously untreatable diseases.

## Figures and Tables

**Table 1:** Summary of key S1R and S2R ligands with indications, mechanisms, and developmental status.

Ligand	Sigma Receptor Target	Disease	Mechanism of Action	Developmental Status	Ref.
AF710B	Sigma 1 Agonist	Alzheimer’s Disease	↓ cognitive impairment ↓ amyloid plaques ↓ inflammatory cytokines	Preclinical	[108,118]
Aniline Derivatives	Sigma 1 Agonist	Stroke	↓ p-PERK and p-IRE1α expression ↓ ER stress	Preclinical	[49]
Afobazole	Sigma 1 Agonist	Stroke	↓ Bax and caspase-3 ↑ Bcl-2 ↓ neuronal death ↓ reactive astrocytes	Preclinical	[55,181]
Blarcamesine	Sigma 1 Agonist	Alzheimer’s Disease, Multiple Sclerosis	MS: ↑ oligodendrogliosis ↓ apoptosis and excitotoxicity ↓ reactive oxygen species and quinolinic acid	Phase IIb/III (Alzheimer’s Disease) Preclinical (Multiple Sclerosis)	[90,136]
Dexmedetomidine	Sigma 1 Agonist	Stroke	↓ BBB permeability ↓ neuronal damage ↓ CHOP, Caspase-3, and JNK ↑ Occludin stabilization	Approved (Sedation)	[48,67,182]
Dipentylammonium	Sigma 1 Agonist	Alzheimer’s Disease	↑ neurite growth length ↓ excitotoxicity ↓ NFkB activation	Preclinical	[126,127]
Donepezil	Sigma 1 Agonist	General Neurodegeneration	↑ oxidative respiration ↑ mitochondrial membrane potentials	Approved (Alzheimer’s Disease)	[183,184]
Fluvoxamine	Sigma 1 Agonist	ALS	↑ chaperone activity ↑ Pom121 expression ↑ TFEB nuclear translocation ↑ LC3-II expression	Preclinical	[143]
N,N-Dimethyltryptamine	Sigma 1 Agonist	Alzheimer’s Disease, Stroke	Alzheimer’s Disease: ↓ cognitive impairment ↓ amyloid plaques ↑ MAM-associated proteins Stroke: ↓ apoptosis and ferroptosis ↓ infarct size ↑ BDNF expression ↓ TNF-α, IL1-β, IL-6	Phase I (Alzheimer’s Disease) Preclinical (Stroke)	[47,53,72,114]
Oxeladin	Sigma 1 Agonist	Stroke	↑ neurologic function **↓** infarct size ↑ BDNF expression	Preclinical	[52]
OZP002	Sigma 1 Agonist	Alzheimer’s Disease	↓ cognitive impairment ↓ reactive oxygen species and lipid peroxidation ↓ Bax, TNFα, IL-6 ↓ reactive gliosis ↑ synaptophysin and choline acetyltransferase	Preclinical	[117]
(+)-Pentazocine	Sigma 1 Agonist	General Neurodegeneration	↓ microglial recruitment ↓ GAD, SOD, and p65	Preclinical	[111]
PRE-084	Sigma 1 Agonist	ALS, Alzheimer’s Disease, Parkinson’s Disease, Stroke	ALS: ↑ neuromuscular function ↑ BiP, EIF2α/ATF4, NRF2 Alzheimer’s Disease: ↑ hippocampal proliferation ↓ reactive gliosis ↓ microglial activation Parkinson’s Disease: ↑ dopaminergic protection ↓ microglial activation ↓ MPTP-induced damage Stroke: ↓ spreading depolarizations ↑ myelin density ↑ CNpase, MOG, NG2 cells, PDGFRα ↓ BBB permeability ↓ learning impairments ↑ BDNF, NR2A, CaMKIV, and TORC1	Preclinical	[58,62,72,116,121,145,165,166,185]
Pridopidine	Sigma 1 Agonist	ALS, Alzheimer’s Disease, Parkinson’s Disease, Huntington’s Disease	ALS: ↑ neuromuscular function ↑ ERK ↓ SOD1 aggregation Alzheimer’s Disease: ↓ excitotoxicity ↑ synapses and dendritic spines ↑ ERK and Akt signaling ↑ long term potentiation Parkinson’s Disease: ↑ dopaminergic protection ↑ GDNF, BDNF, ERK Huntington’s Disease: ↑ BDNF, TrkB, GR, D1R, cAMP ↓ ER stress ↓ reactive oxygen species ↑ calbindin and homer1a	Phase III (Huntington’s Disease) Phase II/III (ALS) Preclinical (Alzheimer’s Disease, Parkinson’s Disease)	[89,94,122,144,149,154,156,158,160,167,169,186]
T-817MA	Sigma 1 Agonist	Alzheimer’s Disease	↓ cognitive impairment ↑ hippocampal proliferation	Phase IIa	[137,187]
TS-157	Sigma 1 Agonist	Stroke	↑ neurite outgrowth ↑ ERK signaling ↑ motor recovery	Preclinical	[64]
Ulinastatin	Sigma 1 Agonist	Stroke	↑ motor recovery	Preclinical	[63]
WLB-87848	Sigma 1 Agonist	Alzheimer’s Disease	↓ cognitive impairment ↑ neuron viability	Preclinical	[125]
BD1063	Sigma 1 Antagonist	ALS	↑ neuromuscular function	Preclinical	[145]
WLB-89462	Sigma 2 Agonist	Alzheimer’s Disease	↓ cognitive impairment	Preclinical	[133]
CT1812	Sigma 2 Antagonist	Alzheimer’s Disease	↓ cognitive impairment ↓ amyloid plaques ↓ phosphorylated tau fragments	Phase II	[132,138,188,189,190]
S1RA	Sigma 2 Antagonist	Stroke	↑ stroke recovery ↓ reactive gliosis ↓ MMP-9 expression	Preclinical	[57]
SAS-0132	Sigma 2 Antagonist	Alzheimer’s Disease	↓ cognitive impairment	Preclinical	[134]

## Data Availability

Not applicable.
